# ERp29 forms a feedback regulation loop with microRNA-135a-5p and promotes progression of colorectal cancer

**DOI:** 10.1038/s41419-021-04252-z

**Published:** 2021-10-19

**Authors:** Jiebin Huang, Mengxia Jing, Xixi Chen, Yuanqi Gao, Huiying Hua, Chun Pan, Jing Wu, Xinqiong Wang, Xuehua Chen, Yujing Gao, Chundi Xu, Pu Li

**Affiliations:** 1 Department of Pediatrics, Ruijin Hospital, Shanghai Jiao Tong University School of Medicine, Ruijin Er Rd.197, Shanghai, 200025 China; 2 Department of Oncology, Zhongshan Hospital, Fudan University, Shanghai, 200032 China; 3 NHC Key Laboratory of Metabolic Cardiovascular Diseases Research, School of Basic Medical Sciences, Ningxia Medical University, Yinchuan, 750004 China

**Keywords:** Cell death, Oncogenes

## Abstract

Expression of endoplasmic reticulum (ER) stress-associated genes is often dysregulated in cancer progression. ER protein 29 (ERp29) is abnormally expressed in many neoplasms and plays an important role in tumorigenesis. Here, we showed ERp29 is a novel target for microRNA-135a-5p (miR-135a-5p) to inhibit the progression of colorectal cancer (CRC); correspondingly, ERp29 acts as an oncoprotein in CRC by promoting proliferation and metastasis of CRC cells, and suppressing apoptosis of the cells. More importantly, we found that miR-135a-5p expression is reversely upregulated by ERp29 through suppressing IL-1β-elicited methylation of miR-135a-5p promoter region, a process for enterocyte to maintain a balance between miR-135a-5p and ERp29 but dysregulated in CRC. Our study reveals a novel feedback regulation loop between miR-135a-5p and ERp29 that is critical for maintaining appropriate level of each of them, but partially imbalanced in CRC, resulting in abnormal expression of miR-135a-5p and ERp29, which further accelerates CRC progression. We provide supporting evidence for ERp29 and miR-135a-5p as potential biomarkers for diagnosis and treatment of CRC.

## Introduction

Colorectal cancer (CRC) is an aggressive primary intestinal malignancy with approximately 1.8 million new diagnoses and 0.88 million deaths per year, being the third leading cause of morbidity and second-highest mortality of all kinds of cancers worldwide [1]. Effective diagnostic methods and available therapeutic options are limited. Therefore, it is imperative to further discover the mechanism underlying CRC progression and identify reliable diagnostic markers and targets for the treatment of CRC.

ERp29 is a novel endoplasmic reticulum protein that is expressed widely in many tissues [2–4], playing an important role in protein unfolding, modification and secretion [5], involved in many pathological conditions, including cancer development [6–9] and age-related degenerate disorders [10]. Ye et al showed that downregulation of ERp29 was commonly found in GC tissues and highly correlated with more aggressive phenotypes and poorer prognosis; knockdown of ERp29 induced epithelial-to-mesenchymal transition in GC cells [11]. ERp29 was also reported to counteract the effect of ER stress (ERS) [12], a process which is known as one of the endoplasmic reticulum quality control systems. Besides having protective effect through inducing the expression of chaperones such as glucose regulatory proteins, ERS also induces endogenous apoptosis of cells independently by activating Bax to antagonize the protective effect of Bcl-2 [13], which also participates in pyroptosis by inhibiting the production of IL-1β [14]. Furthermore, ERp29 expression is upregulated under genotoxicity stress [15–18]. However, it remains largely unknown how the expression of ERp29 is regulated.

MicroRNAs (miRNAs) are one type of small non-coding RNAs of 18-25 nt in length, which mostly inhibit translation and/or negatively regulate the stability of mRNAs by binding to the 3’-untranslated region (3’UTR) of mRNAs [19]. Accumulating data have indicated that miRNAs play an important role in the progression and metastasis of CRC [20–22]. miR-135a-5p has been implicated in many malignant tumors [23–25], including CRC. Previous study reported miR-135a-5p could inhibit the progression and metastasis of CRC cells by regulating SIRT1 [26], which plays an important role in tumorigenicity through p53 signaling pathway [27].

In this study, we demonstrated that miR-135a-5p inhibits in vivo and in vitro growth of CRC by targeting ERp29; correspondingly, ERp29 acts as an oncoprotein in CRC by promoting proliferation and metastasis of CRC cells, and suppressing apoptosis of the cells. More importantly, we found ERp29 could elevate miR-135a-5p expression by decreasing IL-1β-induced methylation of miR-135a-5p promoter region, by which ERp29 forms a feedback regulation loop with miR-135a-5p, and maintains the expression balance between ERp29 and miR-135a-5p; the dysregulation of this loop may lead to CRC progression.

## Results

### miR-135a-5p functions as a tumor suppressor in CRC

**Fig. 1:**
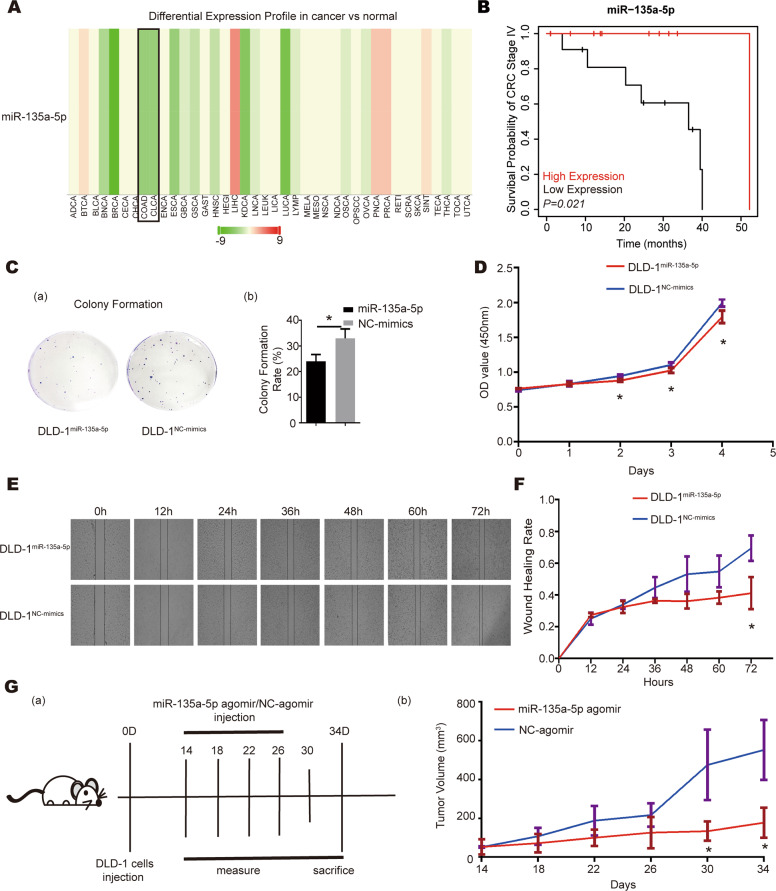
miR-135a-5p functions as a tumor suppressor in CRC. **A** miR-135a-5p was downregulated in most tumor tissues compared to normal tissues based on the result analyzed from dbDEMC database. The horizontal axis displayed the abbreviation of different cancer types (ADCA adrenocortical cancer, BTCA biliary tract cancer, BLCA bladder cancer, BNCA brain cancer, BRCA breast cancer, CECA cervical cancer, CHCA chordoma, COAD colon cancer, CLCA colorectal cancer, ENCA endometrial cancer, ESCA esophageal cancer, GBCA gallbladder carcinoma, GSCA gastric cancer, GAST gastrointestinal stromal tumor, HNSC head and neck cancer, HEGI hemangioma, LIHC liver hepatocellular carcinoma, KDCA kidney cancer, LNCA larynx cancer, LEUK leukemia, LICA liver cancer, LUCA lung cancer, LYMP lymphoma, MELA melanoma, MESO mesothelioma, NSCA nasopharyngeal cancer, NDCA neuroendocrine cancer, OSCA oral squamous cell carcinoma, OPSCC oropharyngeal squamous cell carcinoma, OVCA ovarian cancer, PNCA pancreatic cancer, PRCA prostate cancer, RETI retinoblastoma, SCRA sarcoma, SKCA skin cancer, SINT small intestinal neuroendocrine tumor, TECA testicular cancer, THCA thyroid cancer, TOCA tonsil cancer, UTCA uterus cancer). **B** The survival curve of CRC patients at stage IV (*P* = *0.021*). **C** Plate colony formation validated that miR-135a-5p reduced proliferation of DLD-1 cells; (a) Representative images of plate colony formation; (b) Colony formation rate of DLD-1 cells. **D** CCK8 assay showed that miR-135a-5p inhibited the proliferation ability of DLD-1 cells. **E** Wound-healing assay demonstrated that miR-135a-5p decreased DLD-1 cells migration. **F** Wound-healing rate of DLD-1 cells transfected with miR-135a-5p mimics or NC-mimics. **G** (a) A flow chart depicting the in vivo experimental design; (b) miR-135a-5p inhibited the in vivo growth of DLD-1 cells as showed by growth curve of xenograft tumors. *P < *0.05*.

In order to determine the function of miR-135a-5p during tumorigenesis of CRC, firstly, we compared miR-135a-5p expression levels in different types of tumor tissues to the corresponding normal tissues using the database of dbDEMC3.0 (http://www.biosino.org/dbdemc/) [28]. As shown in Fig. 1A, miR-135a-5p was downregulated in most type of tumor tissues, including colon cancer (COAD) and colorectal cancer (CLCA). Then we analyzed the correlation of miR-135a-5p expression with the prognosis of CRC in Pan-cancer miRNA-Kaplan–Meier plotter [29]. As shown in Fig. 1B, miR-135a-5p expression level was positively correlated with the survival rate of patients with stage IV CRC, the tumor with the highest malignancy and the worst prognosis. The above results indicate that miR-135a-5p might function as a tumor suppressor to inhibit the development and progression of CRC.

To confirm the inhibitory effect of miR-135a-5p on CRC, we altered miR-135a-5p expression by transfecting miR-135a-5p mimics into CRC cells followed by further biological function experiments. CCK-8 assay and plate colony formation assay were used to evaluate cell proliferation; and wound-healing assay was used to assess the ability of cell migration. Notably, overexpression of miR-135a-5p in DLD-1 cells not only reduced colony formation capacity and cell proliferation (Fig. 1C and D), but also slowed down the wound-healing process of the cells (Fig. 1E and F). The consistent results were observed in sw480 and sw1116 CRC cell lines (Supplementary Figure 1). Moreover, the injection of miR-135a-5p agomir into the xenograft tumors of CRC cells in mice could significantly inhibit their growth (Fig. 1G). Taken together, these results indicate miR-135a-5p could inhibit the proliferation and tumorigenesis of CRC.

### ERp29 identifies as a direct target of miR-135a-5p

**Fig. 2:**
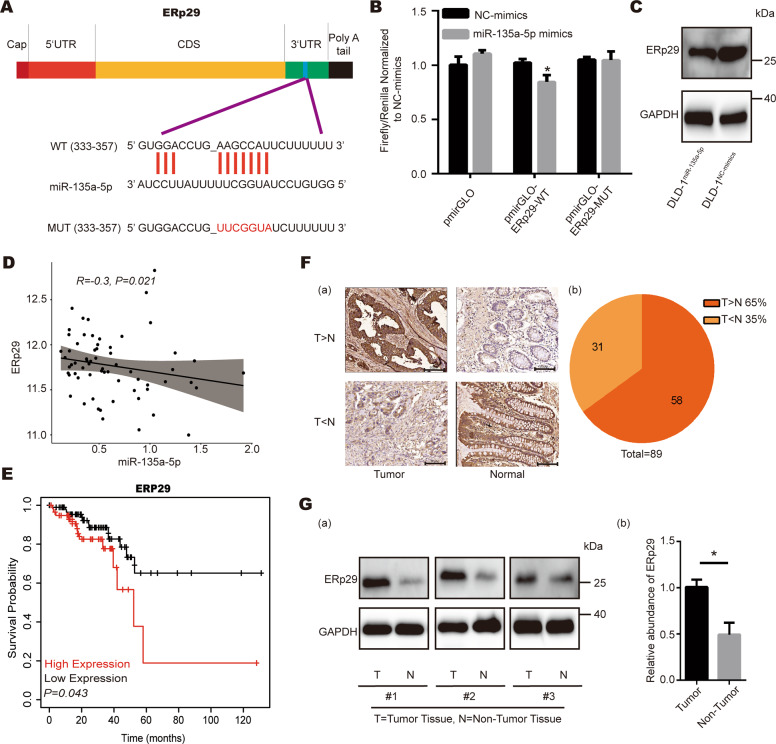
Identification of ERp29 as a direct target of miR-135a-5p during tumorigenesis of CRC. **A** Predicted binding site of miR-135a-5p within the 3ʹ UTR of ERp29 mRNA. The wild-type (WT) and mutant (MUT) 3ʹUTR of ERp29 were constructed into luciferase reporter vector respectively. **B** Luciferase reporter assay confirmed direct recognition of ERp29 3ʹ UTR by miR-135a-5p. **C** Western blot analysis of ERp29 expression in DLD-1 cells transfected with miR-135a-5p mimics or negative control (NC) mimics. **D** ERp29 expression was negatively correlated with miR-135a-5p in spearman correlation analysis (*R* = −0.3, *P* = 0.021). **E** The survival rate of CRC patients was negatively correlated with ERp29 expression (*P* = *0.043*). **F** (a) Expression of ERp29 was detected by IHC staining in CRC tissues and adjacent non-tumor tissues. Original magnification: ×200; (b) Higher expression of ERp29 in tumor tissues compared to adjacent non-tumor tissues accounts for 65% of the total 89 pairs of CRC tissues, while lower expression of ERp29 accounts for 35%. **G** (a) Western blot analysis of ERp29 expression in three pairs of CRC tissues and adjacent non-tumor tissues; (b) Relative abundance of ERp29 in tumor tissues compared with adjacent non-tumor tissues. *P < 0.05.

It is well-known that miRNAs function mainly through suppressing the expression of target genes. To find potential target genes of miR-135a-5p in CRC, we analyzed the predictive results of miR-135a-5p in TargetScan database [30], and noticed that 3’ UTR of ERp29 transcript has a highly conserved binding site for miR-135a-5p (Fig. 2A). Dual luciferase reporter assay was then applied to verify the targeting relationship between miR-135a-5p and ERp29. As shown in Fig. 2B, overexpression of miR-135a-5p resulted in a decrease in the activity of luciferase construct containing ERp29 3’ UTR compared to negative mimics control, while this effect was attenuated when the binding site of miR-135a-5p in ERp29 3’UTR was mutated. We further confirmed ERp29 as a target of miR-135a-5p by detecting the protein level of ERp29, which showed that transfection of miR-135a-5p mimics could markedly reduce ERp29 protein level (Fig. 2C; Supplementary Figure 1A, 1E). Furthermore, Spearman correlation analysis was used to evaluate the relationship between miR-135a-5p and ERp29 in CRC. As shown in Fig. 2D, there was a negative correlation between ERp29 and miR-135a-5p at the expression level (*R* = −0.3, *P* = 0.021).

Then, to further confirm the relationship between ERp29 and miR-135a-5p, we utilized the database of Targetscan, miRcode [31], miRDIP [32], miRWalk [33] and miRmap [34], to predict the miRNAs associated with ERp29 and construct a Venn diagram showing the overlap of the predicted miRNAs. The results indicated that miR-135a-5p and miR-24-3p were the two candidates out from the five databases; however, there was no statistically significant correlation between ERp29 and miR-24a-3p (*P* = 0.69) (Supplementary Figure 2A and 2B). Together, the above results further suggest that ERp29 is a functional direct target of miR-135a-5p with high reliability.

We then evaluated the correlation of ERp29 expression and prognosis of CRC by survival analysis. As shown in Fig. 2E, the survival rate of patients with high ERp29 expression was lower than that of patients with low ERp29 expression during the follow-up, indicating ERp29 is an indicator for poor prognosis of CRC. Next, we determined the expression status of ERp29 in CRC tissues by immunohistochemical staining (IHC) and western blot. Of the 89 pairs of CRC tissues, 65% (58 out of 89) showed higher expression of ERp29 in tumor tissues compared with adjacent non-tumor tissues, while only 35% (31 out of 89) showed downregulation of ERp29 in tumor tissues compared with adjacent non-tumor tissues (Fig. 2F). Western blot results also indicated the protein level of ERp29 in tumor tissues was higher than that in non-tumor tissues (Fig. 2G).

### ERp29 promotes the growth and migration of CRC cells in vitro

**Fig. 3:**
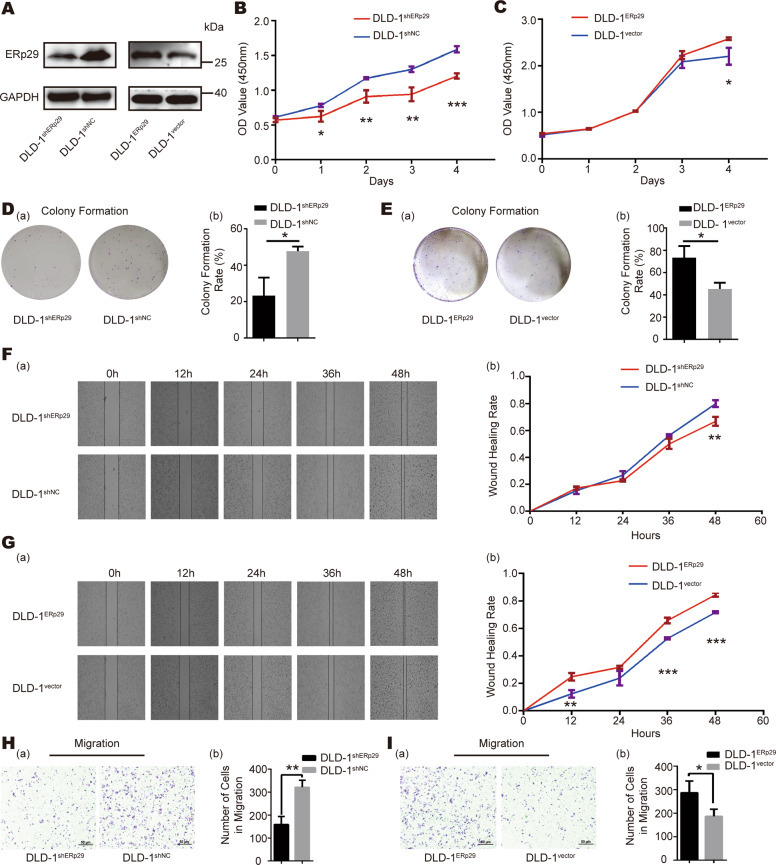
ERp29 promotes proliferation and migration of CRC cells. **A** Western blot analysis was performed to measure ERp29 expression in DLD-1 cells transfected with vectors for overexpressing or knocking down ERp29, or corresponding control vectors. **B** Knockdown of ERp29 inhibited cell proliferation as measured by CCK8 assay. **C** Overexpression of ERp29 promoted cell proliferation as measured by CCK8 assay. **D**, **E** ERp29 promoted the proliferation ability of DLD-1 in plate colony formation assay; (a) Representative images of plate colony formation; (b) Colony formation rate of DLD-1 cells. **F**, **G** Wound-healing assay showed that ERp29 enhanced the migration ability of DLD-1; (a) Representative images of wound-healing assay; (b) Wound-healing rate of DLD-1 cells. **H**, **I** Validation that ERp29 enhances the migration ability of DLD-1 by transwell chamber migration assay; (a) Representative images of transwell chamber migration assay; (b) Number of DLD-1 cells on the lower surface of the chambers. *P < 0.05, **P < 0.01, ***P < 0.001.

Markedly elevated ERp29 expression in CRC tissues prompted us to evaluate the effect of ERp29 on the biological characteristics of CRC cells. ERp29 overexpression and knockdown vectors were constructed and stably transfected into DLD-1 cells respectively, with empty vector as the negative control. The overexpression and knockdown efficiency of ERp29 in the cells were validated by western blot (Fig. 3A). Then the cells were subjected to examination of cell growth and migration abilities. As shown in Fig. 3B and C, compared with the control group (DLD-1^shNC^ or DLD-1^vector^), downregulation of ERp29 inhibited the proliferation of DLD-1 cells, whereas upregulation of ERp29 promoted cell proliferation. To further determine the effect of ERp29 on cell growth, we performed plate colony formation assay, which revealed that cells with ERp29 knockdown formed less colonies than the control cells (Fig. 3D), while overexpression of ERp29 exhibited the opposite effect (Fig. 3E).

Wound-healing assay and transwell migration assay were performed to evaluate the effect of ERp29 on migration ability of DLD-1 cells. As shown in Fig. 3F, compared with control cells (DLD-1^shNC^), ERp29 knockdown (DLD-1^shERp29^) cells healed the wound at a relatively slower speed. Conversely, the wound nearly closed at 48 h post-scratch in ERp29 overexpression cells, whereas control cells failed to heal the wound at the same time point (Fig. 3G). Transwell assay showed that DLD-1^shERp29^ cells migrating through the chamber were fewer than control cells (Fig. 3H); whereas, DLD-1 cells with ERp29 overexpression had higher migration ability compared with control cells (Fig. 3I). The promoting effects of ERp29 on cell proliferation and migration of CRC cells were further validated in another two CRC cell lines, sw480 and sw1116 (Supplementary Figure 3).

### ERp29 enhances in vivo tumorigenicity and metastasis of CRC cells

**Fig. 4:**
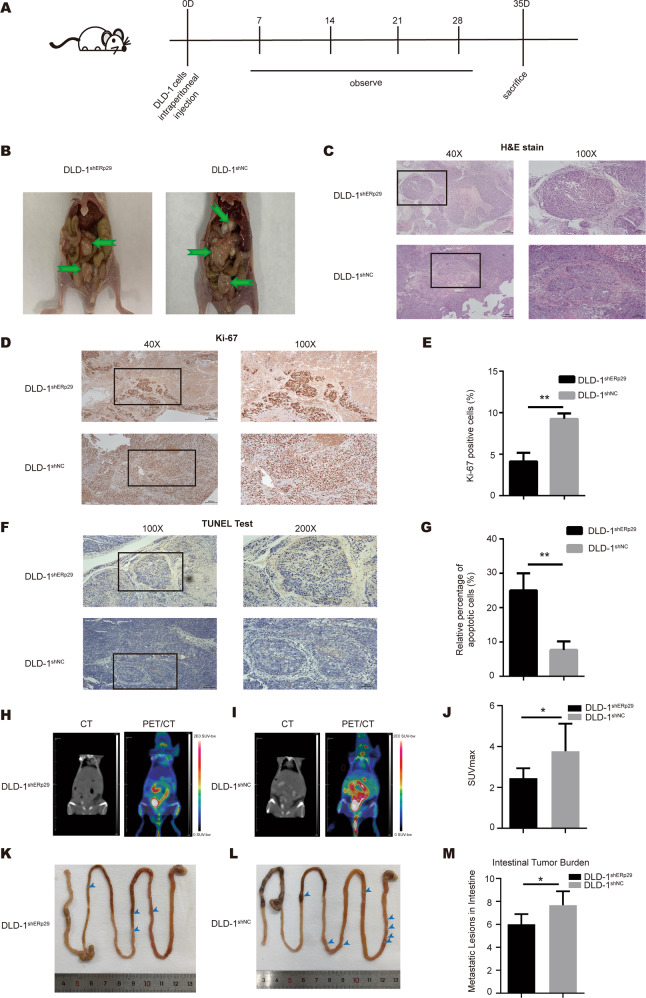
ERp29 stimulates metastasis of CRC in vivo. **A** A flow chart depicting the in vivo experimental design. **B** Photographs of tumor nodules in abdominal cavity metastasis model. **C** Representative H&E stain images of tumor nodules. **D** Expression of Ki-67 in the tumor nodules was measured by IHC staining. Tumor nodules formed from DLD-1^shERp29^ cells had lower expression of Ki-67 compared with that from DLD-1^shNC^ cells. **E** Relative percentage of Ki-67 positive cells in tumor nodules. **F** Representative images of TUNEL assay for tumor nodules. Tumor nodules formed from DLD-1^shERp29^ cells had more apoptotic cells than those from DLD-1^shNC^ cells. **G** Relative percentage of apoptotic cells in tumor nodules. **H** Representative CT and PET/CT images of the DLD-1^shERp29^ cells-bearing mice. **I** Representative CT and PET/CT images of the DLD-1^shNC^ cells-bearing mice. **J** The SUVmax value of the abdominal organs in the DLD-1^shERp29^ and DLD-1^shNC^ groups. **K** Representative photo of the complete gastrointestinal tract of DLD-1^shERp29^ cells-bearing mice. **L** Representative photo of the complete gastrointestinal tract of DLD-1^shNC^ cells-bearing mice. **M** Quantitative display of the metastatic lesions in the DLD-1^shERp29^ and DLD-1^shNC^ groups. *P < 0.05, **P < 0.01.

We proceeded to investigate the role of ERp29 in tumorigenicity using abdominal cavity metastasis model of nude mouse. After three days of pre-feeding to adapt to the environment, nude mice were injected with suspension of DLD-1 cells with stable knockdown of ERp29 expression (DLD-1^shERp29^), or the same amount of control cells suspension (DLD-1^shNC^) (Fig. 4A). As shown in Fig. 4B, the number and volume of tumor nodules formed from DLD-1^shERp29^ cells were less than that from DLD-1^shNC^ cells. The tumor nodules were verified by H&E stain (Fig. 4C), and Ki-67 expression levels in the tumor nodules were then examined by IHC. As shown in Fig. 4D and E, tumor nodules formed from DLD-1^shERp29^ cells had lower expression of Ki-67 compared with that from DLD-1^shNC^ cells, indicating a lower proliferation capacity of DLD-1 cells with low ERp29 expression.

Furthermore, we established orthotopic xenograft mouse model, which could better simulate the tumor metastasis. The nude mice were randomly divided into two groups, and DLD-1^shERp29^ or DLD-1^shNC^ cells were injected into the cecal wall of corresponding mice respectively through laparotomy (Supplementary Figure 4). Representative CT and ^18^F-FDG PET/CT images of mice with high (DLD-1^shNC^ group) or low (DLD-1^shERp29^ group) accumulation of ^18^F-FDG in the intestine were shown in Fig. 4H and I. We evaluated ^18^F-FDG accumulation through SUVmax, a widely used semiquantitative measurement. The value of SUVmax in the abdomen of DLD-1^shERp29^ group was significantly lower than that in the control group (Fig. 4J). Meanwhile, the complete gastrointestinal tracts were photographed (Fig. 4K and L) and the metastatic lesions were counted. Consistently, the mice of DLD-1^shERp29^ group had less metastatic lesions than DLD-1^shNC^ group mice (Fig. 4M).

Overall, based on the results of in vitro and in vivo experiments, we concluded ERp29 plays a role in facilitating tumorigenicity and metastasis in CRC.

### ERp29 inhibits apoptosis of CRC cells

Given that suppressed apoptosis is one of the hallmarks of malignant tumors [35], we evaluated the apoptotic situation of tumor nodules obtained in the abdominal cavity metastasis model using TUNEL test. The results showed that there were more apoptotic cells in tumor tissues formed from DLD-1^shERp29^ cells than that from control DLD-1^shNC^ cells (Fig. 4F and G), suggesting ERp29 may inhibit apoptosis of DLD-1 cells.

**Fig. 5:**
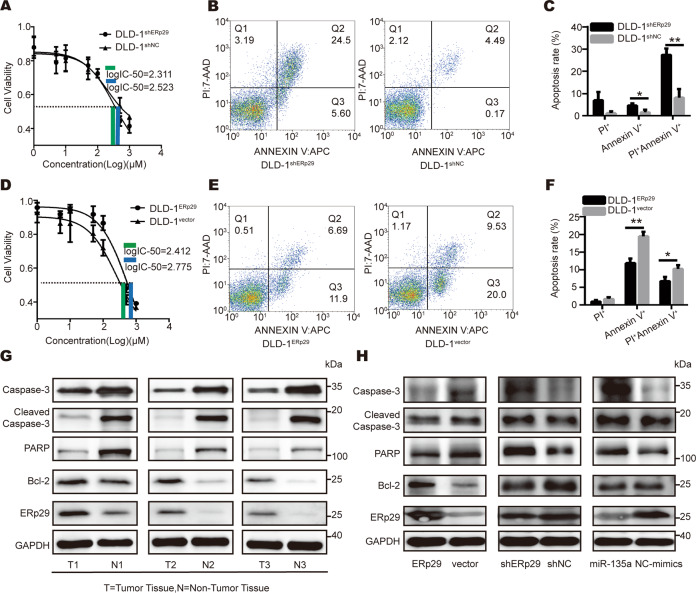
ERp29 inhibits apoptosis of CRC cells. **A** Cytotoxicity of 5-FU to DLD-1^shERp29^ and DLD-1^shNC^ cell lines were measured by CCK8 assay, and log IC50 for the cells were calculated. **B** Flow cytometry analysis was performed to test 5-FU-induced apoptosis in DLD-1^shERp29^ and DLD-1^shNC^ cell lines. **C** The percentage of apoptotic cells in DLD-1^shERp29^ and DLD-1^shNC^ group. **D** Cytotoxicity of 5-FU to DLD-1^ERp29^ and DLD-1^vector^ cell lines were measured by CCK8 assay, and log IC50 for the cells were calculated. **E** Flow cytometry analysis was performed to test 5-FU-induced apoptosis in DLD-1^ERp29^ and DLD-1^vector^ cell lines. **F** The percentage of apoptotic cells in DLD-1^ERp29^ and DLD-1^vector^ group. **G** Comparison of protein levels of ERp29 and several apoptosis-related proteins in CRC tissues and adjacent normal tissues. **H** Protein levels of the apoptosis-related proteins were tested by western blot after expression of ERp29 or miR-135a-5p was altered in DLD-1 cells. *P < 0.05, **P < 0.01.

Anti-apoptosis ability confers tumor cells chemoresistance to chemotherapeutic drugs [36], such as 5-FU [37]. To test the effect of ERp29 on chemoresistance of CRC cells, we induced cell apoptosis with 5-FU in the DLD-1 cells with different expression levels of ERp29, and performed CCK-8 assay to measure the toxicity of 5-FU to the cells. As shown in Fig. 5A and D, DLD-1 cells with ERp29 knockdown had a lower log IC50 (half maximal inhibitory concentration) compared to control shNC cells (2.311 μM vs 2.523 μM); while DLD-1 cells with overexpression of ERp29 had a higher log IC50 (2.775 μM) compared with control cells (2.412 μM), indicating that ERp29 could decrease the sensitivity to 5-FU in DLD-1 cells. Moreover, we measured cell apoptosis using flow cytometry, which showed ERp29 knockdown could increase the ratio of apoptotic cells induced by 5-FU, whereas overexpression of ERp29 in the cells reduced the ratio of apoptotic cells (Fig. 5B, C, E and F).

Next, we investigated how ERp29 inhibits apoptosis of CRC cells. We detected levels of several key apoptosis-related proteins by immunoblotting in surgical CRC specimens with matched adjacent normal tissues, and found that the expression of ERp29 and the anti-apoptotic protein Bcl-2 were upregulated in tumor tissues in comparison to normal tissues; while apoptosis markers, including cleaved caspase-3 and PARP, were downregulated in tumor tissues compared with normal tissues (Fig. 5G). We further examined the effects of ERp29 on the expression of these apoptosis-related proteins in DLD-1 cells. As expected, overexpression of ERp29 could decrease protein levels of cleaved caspase-3 and PARP, but increase Bcl-2 protein level. Conversely, knockdown of ERp29 showed opposite results. As a negative regulator for ERp29, miR-135a-5p had a similar effect with knockdown of ERp29 (Fig. 5H).

Collectively, these findings suggest that ERp29 might inhibit the activation of cell apoptotic signal, which contributes to the development of CRC.

### ERp29 upregulates expression of miR-135a-5p by downregulating IL-1β

**Fig. 6:**
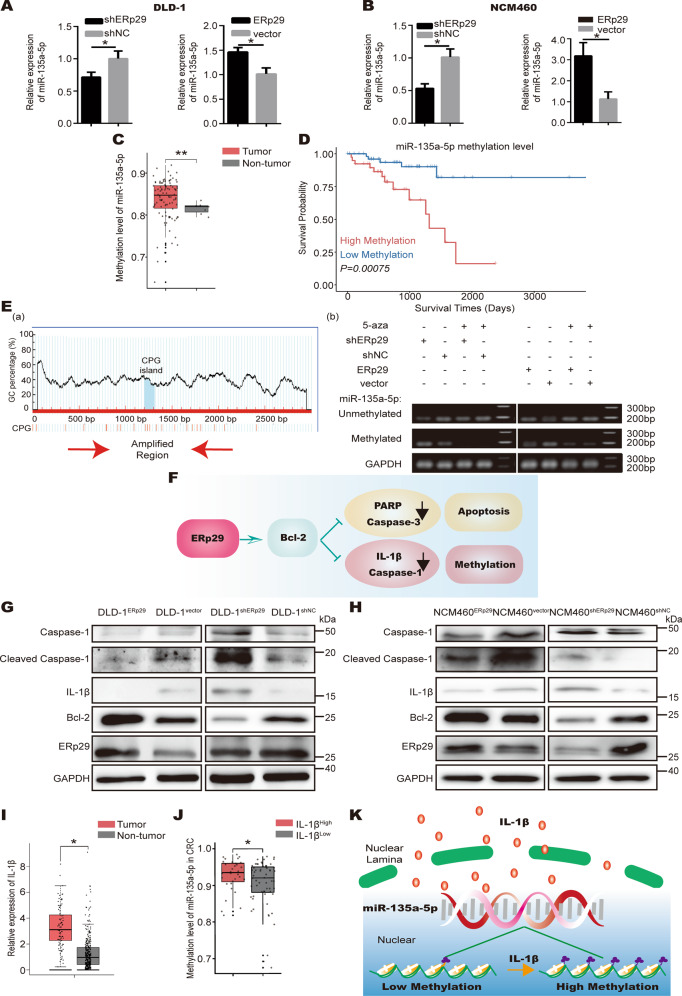
ERp29 upregulates miR-135a-5p expression by downregulating IL-1β-induced DNA methylation. **A** qRT-PCR analysis of miR-135a-5p levels in DLD-1 cells transfected with ERp29-expressing vector, shERp29 vector, or the negative control vector. **B** qRT-PCR analysis of miR-135a-5p levels in NCM460 cells transfected with ERp29-expressing vector, shERp29 vector, or the negative control vector. **C** The methylation level of miR-135a-5p in CRC tissues was higher than non-tumor tissues. **D** The survival rate of CRC patients was negatively correlated with promoter methylation level of miR-135a-5p (*P* = 0.00075). **E** MSP analysis of miR-135a-5p promoter methylation level. (a) CPG island in the miR-135a-5p promoter region and the region of primer designed for MSP analysis; (b) Promoter methylation level of miR-135a-5p was measured by MSP analysis in DLD-1 cells transfected with ERp29-expressing vector, shERp29 vector, or the negative control vector, with or without 5-AZA treatment. **F** A diagram showing how ERp29 affects apoptosis and methylation. **G** Western blot analysis of protein levels of Caspase-1, Cleaved-caspase-1, IL-1β, Bcl-2, and ERp29 in DLD-1 cells and (**H**) NCM460 cells transfected with shERp29 vector, ERp29-expressing vector, or the negative control vector. **I** IL-1β was upregulated in CRC tissues (*n* = 275) compared to normal tissues (*n* = 349). **J** The methylation level of miR-135a-5p in high IL-1β expression group (*n* = 32) was higher than low IL-1β expression group (*n* = 60). **K** A diagram showing IL-1β induces methylation in miR-135a-5p promoter region. *P < 0.05, **P < 0.01.

As shown above, ERp29 is a target of miR-135a-5p. Surprisingly, we found ERp29 could reversely influence the expression level of miR-135a-5p. As shown in Fig. 6A, the expression of miR-135a-5p was decreased when ERp29 was knocked down in the cells, while overexpression of ERp29 could increase miR-135a-5p level in the cells. Previous reports showed ERp29 could regulate the expression of downstream protein through DNA methylation [38], and miR-135a-5p expression can be modulated by its promoter methylation status in cancer cells [39]. We then questioned whether ERp29 regulates the expression of miR-135a-5p by modulating its promoter methylation level. Firstly, we investigated the methylation status of miR-135a-5p in CRC using TCGA dataset, which showed a higher miR-135a-5p methylation status in CRC tissues than non-tumor tissues (Fig. 6C). Further survival analysis revealed that high methylation of miR-135a-5p promoter region was inversely correlated with prognosis of CRC patients (Fig. 6D). These *in silico* analyses indicate DNA methylation might be a modulator of miR-135a-5p expression in CRC. We then proceeded to analyze the effect of ERp29 on miR-135a-5p promoter methylation. Methylation level of miR-135a-5p promoter region was detected by methylation-specific PCR (MSP) after ERp29 expression was altered. As shown in Fig. 6E, compared with the corresponding negative control cells, the promoter methylation level of miR-135a-5p was reduced in ERp29 overexpressing DLD-1 cells, while it was increased in ERp29 knockdown cells; in addition, 5-Azacytidine (5-AZA), an inhibitor of DNA methyltransferases [40], could reverse ERp29 knockdown-induced promoter methylation of miR-135a-5p to a certain degree.

**Fig. 7:**
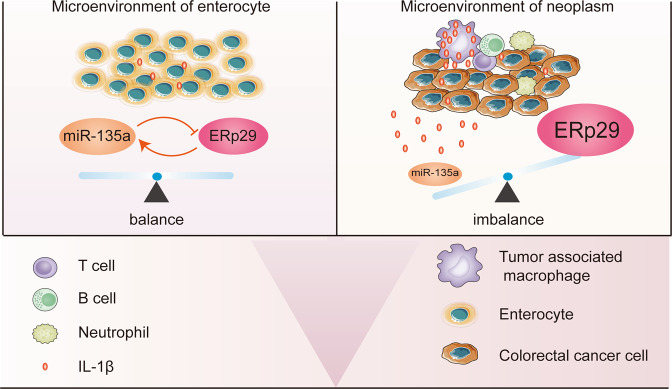
The mechanism for feedback regulation between miR-135a-5p and ERp29 in normal and neoplasm tissues. The feedback loop between miR-135a-5p and ERp29 may function in normal enterocyte while malfunction in CRC due to dramatically increased production of IL-1β by inflammatory cells in the tumor microenvironment.

We then investigated the mechanisms by which ERp29 regulates methylation of miR-135a-5p. As we have confirmed ERp29 could upregulate Bcl-2 expression, and Bcl-2 was reported to inhibit the stimulation of IL-1β [14]. More importantly, IL-1β is one of many inflammatory cytokines produced following caspase-1 activation during pyroptosis [41], and several reports reported that IL-1β could induce DNA methylation [42–45] (Fig. 6F). Therefore, we assumed that ERp29 influences the promoter methylation of miR-135a-5p through IL-1β (Fig. 6K). Accordingly, we examined the production of IL-1β and activation of caspase-1 in DLD-1 cells after ERp29 expression was altered. As shown in Fig. 6G, both IL-1β production and caspase-1 activation were reduced in DLD-1 cells with ERp29 overexpression in comparison to control cells, while knockdown of ERp29 could elevate IL-1β production and caspase-1 activation in the cells. Therefore, as a direct target gene of miR-135a-5p, ERp29 forms a regulation loop with miR-135a-5p by modulating its expression via DNA methylation. However, this regulation loop could not explain the simultaneous existence of miR-135a-5p downregulation and ERp29 upregulation in CRC that we have confirmed above. Considering tumor development is closely accompanied with increased inflammation, we rationalized that ERp29/miR-135a-5p regulation loop may be critical for maintaining their levels in physiologic status; but in CRC, inflammatory cells in the tumor microenvironment produce much more IL-1β robustly, and thereby impair the regulation loop (Fig. 7).

To confirm this, we first detected changes in miR-135a-5p level, IL-1β production and caspase-1 activation in normal enterocyte NCM460 after ERp29 expression was altered. The results were consistent with those in tumor cells, where ERp29 upregulated miR-135a-5p expression and downregulated levels of IL-1β and active form of caspase-1 (Fig. 6B and H), suggesting that this mechanism is generalized but not cancer specific. Next, we investigated IL-1β expression in CRC and non-tumor tissues using TCGA database, which verified dramatic elevation of IL-1β expression in CRC (Fig. 6I); and consistently, the methylation level of miR-135a-5p was higher in the IL-1β^high^ group (Fig. 6J).

Overall, the aforementioned results unveil a feedback loop that ERp29, as a target gene of miR-135a-5p, in turn regulates miR-135a-5p expression via IL-1β-induced methylation of miR-135a-5p promoter region. In normal enterocyte, this feedback loop keeps a balance between miR-135a-5p and ERp29 because of the limited production of autocrine IL-1β. However, in pathophysiological statuses that from ongoing inflammatory states to CRC, the feedback loop malfunctions due to the excess of paracrine IL-1β produced by tumor microenvironmental cells, such as tumor-associated macrophages. Consequently, the balance between miR-135a-5p and ERp29 isdisrupted, which further facilitates disease progression (Fig. 7).

## Discussion

Surgical operation is the most effective treatment for CRC so far [46]. However, due to the lack of early diagnostic methods, vast amounts of CRC patients are diagnosed at a late stage [47, 48]. Thus, many patients lose the opportunity for radical resection because of metastasis, which accounts for the low five-year survival rate [46, 49]. Controlling tumor metastasis is therefore as an important way to extend the 5-year survival rate of patients with CRC and thus improve the quality of life. However, the molecular and regulatory mechanisms of CRC metastasis remain unclear. Therefore, elaborating the molecular mechanism of the occurrence, development, and metastasis of CRC based on the cellular and molecular levels may open up new possibilities for therapeutic strategy.

Non-coding RNAs cover a large part of the human transcriptome [50]. As one member of non-coding RNAs, miRNA plays a complex role in gene expression and is closely related to tumorigenesis. miR-135a-5p reportedly inhibits tumorigenesis and development in most cancers, such as GC [51], esophageal cancer [52], and glioma [53]. In the current study, we found miR-135a-5p expression is positively correlated with the favorable prognosis of CRC. In addition, we confirmed miR-135a-5p could inhibit the proliferation and migration of CRC cells, indicating miR-135a-5p acts as a tumor suppressor in CRC.

Based on the predictive result of bioinformatics analysis, we found miR-135a-5p could target ERp29 mRNA with a high probability of preferential conservation; and we testified this prediction in DLD-1 cells. ERp29 is a novel endoplasmic reticulum protein with its mechanism in tumors unclear. Previous study showed that ERp29 plays a role in inhibiting tumor proliferation by affecting the epithelial-mesenchymal transition mechanism [11], but others reported ERp29 could promote tumor proliferation by counteracting the effect of endoplasmic reticulum stress [12]. In our study, we identified ERp29 as a target gene of miR-135a-5p in regulating CRC development. The results showed that ERp29 promotes the proliferation and migration of CRC cells in vitro and in vivo. Furthermore, we found ERp29 inhibits apoptosis of CRC cells through Bcl-2-suppressed activation of caspase-3 and PARP expression.

More importantly, we found ERp29 suppresses the activation of caspase-1, leading to the inhibition of IL-1β production, which is proposed to boost DNA methylation and result in suppressed expression of target genes [42–45]. Inhibition of caspase-1 activation and IL-1β production by ERp29 may be mediated by Bcl-2, since ERp29 could facilitate Bcl-2 expression which was reported to control IL-1β production via NALP1 [14]. Furthermore, IL-1β was found to be elevated in CRC tumors [54], which was mainly secreted by tumor-associated macrophages [55]. Therefore, ERp29 increases miR-135a-5p expression via reducing IL-1β-induced methylation on the miR-135a-5p promoter region, and forms a regulation loop with miR-135a-5p, a mechanism by which normal cells maintain a balance between miR-135a-5p and ERp29 to prevent excessive growth, but maybe dysregulate in cancer cells owing to a robust increase in exogenous IL-1β produced by inflammatory cells in the microenvironment of CRC.

In conclusion, our study reveals that miR-135a-5p functions as a tumor suppressor by targeting ERp29, which in turn affects proliferation, metastasis, and apoptosis of CRC cells. These findings indicate that miR-135a-5p and ERp29 may be used as potential diagnostic markers, and modulating their expression might be a promising strategy for CRC treatment. In addition, we found a novel regulation loop between ERp29 and miR-135a-5p, clarifying the deregulation of this regulation loop in CRC cells and the underlying mechanisms in the future will be helpful to in-depth understand the mechanism of CRC development.

## Materials and methods

### Human tissue

CRC tissues and paired adjacent non-tumor tissues were obtained from patients who undergo radical resection at the Department of Surgery, Ruijin Hospital, Shanghai, China. All tissues were histologically confirmed, and frozen in liquid nitrogen immediately after the resection of tumor, and stored at ultra-low temperature freezer.

### Cell lines and cell culture

Normal colon mucosa epithelial cell line NCM460 and CRC cell lines DLD-1, sw480, and sw1116 were cultured in RPMI1640 medium supplemented with 10% fetal bovine serum and 1% penicillin-streptomycin at 37 °C in a humidified incubator with 5% CO_2_, as well as 293 T cells, which were all purchased from Shanghai Institutes for Biological Sciences, Chinese Academy of Sciences (Shanghai, China).

### Plasmid construction and transfection

ERp29 cDNA was cloned into the GV492 vector which was transfected into CRC cells using lipofectamine2000 (Invitrogen, California, USA), according to the manufacturer’s instructions. ERp29 shRNA cloned into pGPU6-GFP-Neo plasmid (Gene Pharma, Shanghai, China) was used for silence of ERp29 in CRC cells. 100 pmol of miR-135a-5p mimics were used to overexpress miR-135a-5p, with NC-mimics as negative control. Cells were harvested at 24 h or 48 h after transfection for extracting total RNA or protein.

### Tissue microarray and immunohistochemical analysis

CRC tissue arrays were purchased from the National Engineering Center for BioChips in Shanghai, China. Monoclonal antibody against ERp29 (Abcam, Massachusetts, USA) was used to detect the expression of ERp29 in CRC tissues at a dilution of 1:300. The slides were scanned by Pannoramic MIDI, and calculated the histochemistry score (H-SCORE) using the following formula: H-SCORE = ∑(PI × I) = (percentage of cells of weak intensity × 1) + (percentage of cells of weak intensity × 1) + (percentage of cells of moderate intensity × 2) + percentage of cells of strong intensity × 3); PI stands for the percentage of positive cells to the total number of cells in the section.

### RNA isolation and quantitative realtime-PCR (qRT-PCR)

Total RNA was extracted using RNAiso plus (Takara, Kusatsu, Japan) according to the manufacturer’s protocol. RNA was reversely transcripted into cDNA using GoScript ™ Reverse Transcription Kit (Promega, Wisconsin, USA), and TB Green Premix Ex Taq (Tli RNaseH Plus) (Takara) was then used for qRT-PCR to quantitate miRNA and mRNA levels. Relative levels of mRNA and miRNA were calculated via 2^−ΔΔCt^ method, with GAPDH and U6 as the internal controls respectively.

### Luciferase reporter assay

The reporter plasmids pmirGLO-ERp29-3ʹUTR-WT and pmirGLO-ERp29-3ʹUTR-MUT were synthesized by Shanghai Sangon Biotech company (Shanghai, China). For the luciferase reporter assay, 500 ng reporter plasmid vector, and 100pmol of miR-135a-5p mimics or NC-mimics were transfected into 293 T cells. Luciferase activities were analyzed using a Dual Luciferase Assay Kit (Promega) 24 h after transfection.

### Cell proliferation assay

The proliferation ability of CRC cells was determined by CCK8 assay and plate colony formation assay. For CCK8 assay, 1 × 10^3^ cells were cultured in 96-well plates and incubated for five days; OD450nm was measured 2.5 h after adding 10 μL of CCK8 reagent. For plate colony formation assay, cells were resuspended in RPMI1640 complete medium and seeded onto six-well plates; after incubation for 1–2 weeks, the cells were formalin-fixed and stained with 0.4% crystal violet; colonies were photographed and counted.

### Cell migration assay

Wound-healing assay and transwell chamber migration assay were used to determine the migratory capacity of CRC cells. In the wound-healing assay, 5 × 10^5^ cells were seeded into six-well plate and cultured to 100% confluence, a sterilized 10 μL pipette tip was used to make a straight scratch in the wells subsequently. The cell layers were imaged and migration activity was monitored every 12 h. For the transwell chamber migration assay, the lower chamber was inserted into the wells of 24-well plate which filled with 500 μL medium supplemented with 10% FBS; and cells (1–3 × 10^5^) in 300 μL serum-free medium were seeded in the upper compartment of the chamber. After incubation for 24 h, cells on the upper surface were scraped off with a cotton swab. The migrated cells on the lower surface were fixed in 4% paraformaldehyde, stained with 0.4% crystal violet, photographed, and counted from five random fields under the microscope.

### Western blot analysis

Cells and tissues were lysed in RIPA buffer (Beyotime, Shanghai, China) containing protease inhibitor and phosphatase inhibitor. An equal amount of total proteins were separated by 10% SDS-PAGE gels and transferred to polyvinylidene fluoride (PVDF) membranes. The membranes were blocked with 5% skimmed milk powder for 1 h at room temperature, and then incubated with primary antibody at 4°C overnight. Next day, the membranes were incubated with the secondary antibody at room temperature, and visualized with an enhanced chemiluminescence method. Primary antibodies used for western blot assay were anti-ERp29 (1:1000, Abcam), anti-Bcl-2 (1:1000, CST, Massachusetts, USA), anti-caspase-3 (1:1000, CST), anti-cleaved caspase-3 (1:1000, Absin, Shanghai, China), anti-PARP (1:1000, CST), anti-IL-1β (1:1000, CST), anti-caspase-1 (1:1000, CST) and anti-GAPDH (1:2000, Servicebio, Wuhan, China). GAPDH was used as a reference protein for normalization.

### Flow cytometry

After 24 h of 5-FU treatment (100 μg/mL), cells were collected and incubated with Annexin V-APC (BD, San Diego, USA) and PI-7-AAD (BD) at room temperature for 15 min, and analyzed by FACS subsequently.

### Tumor xenograft models

Thymus-null BALB/c nude mice (male, 4weeks), SPF (specific-pathogen-free) mice, which were purchased from Vital River Laboratory Animal Technology of Beijing (Beijing, China), were pre-fed for three days to adapt to the operating environment. For subcutaneous xenograft model, DLD-1 cells (4 × 10^6^) in 100 μL of PBS were subcutaneously injected into mice, which were randomly selected. After tumor formed in the mice, 1 nmol of miR-135a-5p agomir or NC-agomir were injected into the tumor nodules every four days. The diameters of each tumor nodule including the length (L) and width (W) were measured by the vernier calipers every four days; and the volume was calculated using the formula: (W + L)/2 × W × L × 0.5236. For abdominal xenograft model, ERp29 knockdown cells (4 × 10^6^) were injected into abdominal cavity. After five weeks, the nude mice were sacrificed to collect the tumor nodules for H&E stain, immunohistochemistry and TUNEL test. All animal experiments were conducted following the institutional ethical guidelines on animal care and approved by the Department of Animal Experimentation of the Shanghai Jiao Tong University School of Medicine.

### Orthotopic xenograft mouse model

Thymus-null BALB/c nude mice (male, four weeks) were purchased from Shanghai SLAC Laboratory Animal Company and randomly divided into two groups, respectively as DLD-1^shERp29^ and DLD-1^shNC^. The model was established in the mice according to previous reports and protocols [56–59]. The mice were anesthetized by intraperitoneal injection of avertin (400 mg/kg), then the surgical site was disinfected with iso-betadine and surrounded by sterile drapes. Abdominal access was attained via a one-cm incision through the abdominal wall musculature and peritoneal wall. The caecum was exteriorized and lavaged with sterile saline. Cells (1 × 10^7^) in 50 μL of PBS were slowly injected between the mucosa and muscularis externa layers of the caecal wall. Finally, the caecum was returned to the abdominal cavity, and skin, muscles, and peritoneum were sealed with non-continuous sutures. Penicillin was injected intraperitoneally for three consecutive days after the operation to prevent infection, during which period they were monitored closely for signs of inflammation or infection. Eight weeks after cell injection, the mice were observed through PET/CT; intestinal metastatic lesions were counted, and metastatic index was calculated.

### Micro PET/CT imaging

Prior to micro PET/CT imaging, the mice were fasted overnight (16 h) to minimize bowel movement and maximize tumor ^18^F-FDG uptake. Nude mice were pre-anesthetized with 3% isoflurane in oxygen, injected with 3.7 MBq ^18^F-FDG via the tail vein catheter, and maintained with 1–2% isoflurane on a heated (37 °C) pad. After the 50-minute uptake period, mouse was fixed in the center of the scanning bed in the prone position under continuous anesthesia with 1.5% isoflurane. PET/CT imaging was performed on an Inveon system (Siemens Preclinical Solutions). After scanning, the filtered back projection method reconstructed coronal, cross-sectional, and sagittal tomographic images for analysis. Siemens Invenon Research Workplace (IRW3.0) was used to obtain the caecum site and quantitatively analyze the reconstructed image to obtain the SUVmax value.

### Methylation-specific PCR (MSP)

Genomic DNA was purified from cells and followed by bisulfite treatment using the following steps. Firstly, DNA (2 μg) was denatured by NaOH (3 mM) at 42 °C for 30 min. Freshly prepared hydroquinone (10 mM) and sodium bisulfite (3.6 mM) were then added into the denatured DNA, mixed well, and incubated with 200 μL paraffin oil at 50 °C for 16 h, in the dark. Finally, modified DNA was purified according to the manufacture’s instruction (TIANGEN, Beijing, China). MSP primers were designed around transcription start site of gene sequence using MethPrimer tools [60]. The reactions included 35 cycles of 98 °C for 10 s, 52 °C for 30 s, and 72 °C for 30 s. The final PCR products were detected by agarose gel electrophoresis.

### TCGA data

RNA sequencing data, DNA methylation data, and clinical data for Rectum Adenocarcinoma (READ) patients from TCGA pan-cancer Atlas were obtained from UCSC Xena database [61]. R software (version 4.0.3) with build-in packages and custom routines was used for data analysis. Survival analysis was conducted by R. The methylation level of miR-135a-5p was sorted, with the mean value was used as the cut-off value for defining low- or high- methylation level.

### Kaplan–Meier survival curve analysis

Pan-cancer miRNA-Kaplan–Meier plotter and Pan-cancer mRNA-Kaplan–Meier plotter were conducted through the website (http://kmplot.com/analysis/). The option called “split patients by auto select best cutoff” was selected for defining the expression status of miR-135a-5p or ERp29; less than the cutoff value was considered as low expression, while higher than the cutoff value was defined as high expression.

### Statistical analysis

All the data from at least three independent experiments were graphically presented as mean ± standard deviation. Chi-square test was used for statistical analysis of the frequency between two groups, and *t*-test was used for analyzing statistical difference between two independent samples, in which *P* < 0.05 was considered as statistically significant.

## Supplementary information

Supplementary Material

## Data Availability

The datasets supporting the conclusions of this article are included within the article and its additional files.
