# Supplementary material for: ERp29 forms a feedback regulation loop with microRNA-135a-5p and promotes progression of colorectal cancer
Source: Cell Death Dis. 2021 Oct 19;12(11):965. doi: 10.1038/s41419-021-04252-z (PMC8526686; doi:10.1038/s41419-021-04252-z)
Supplement: Supplementary file 1 — Supplementary Material [file 41419_2021_4252_MOESM1_ESM.docx]

**Supplementary Figure 1**

**
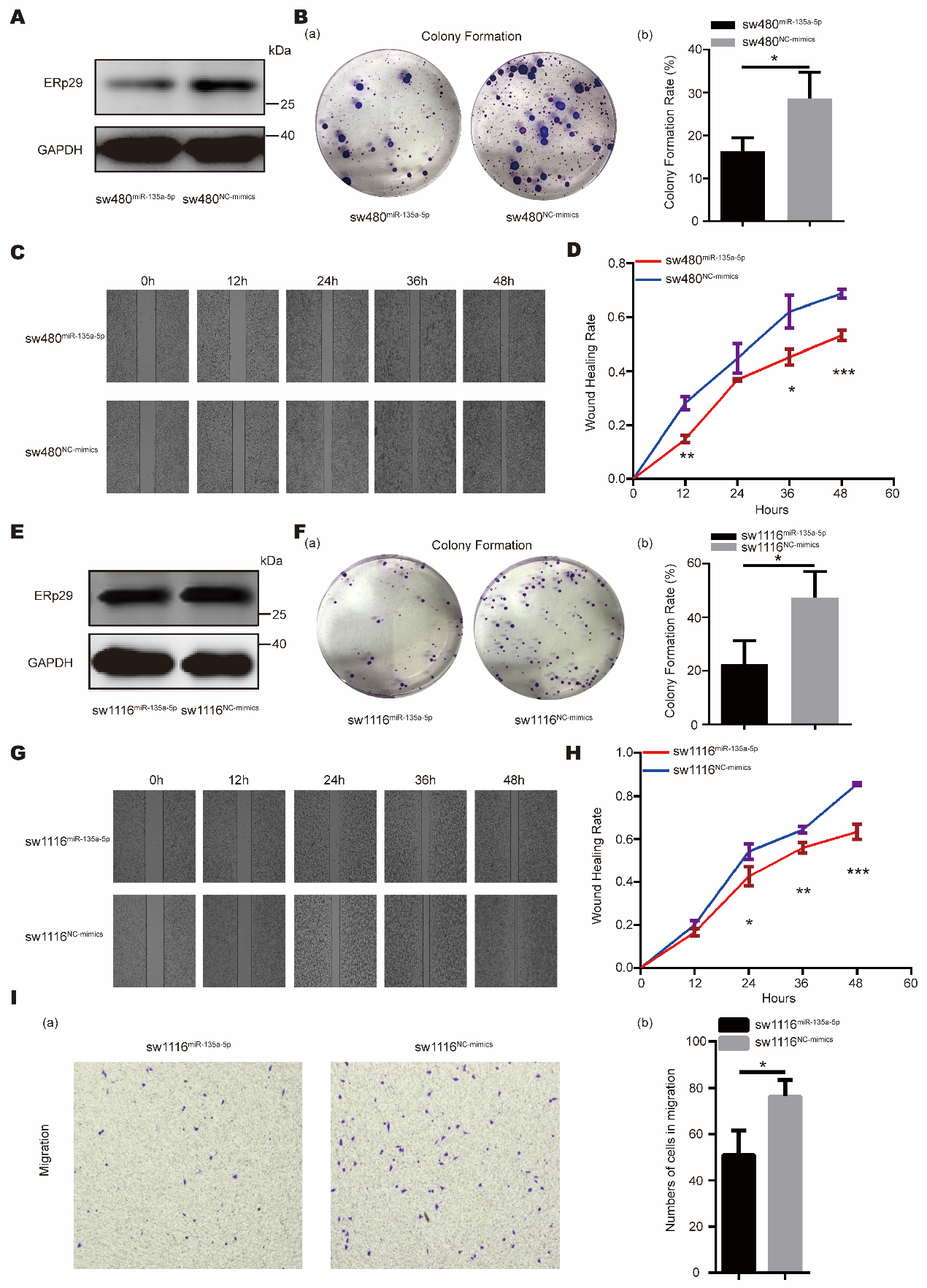
**

**Supplementary Figure 1. miR-135a-5p inhibits proliferation and migration of CRC cells**

(A) ERp29 was downregulated by miR-135a-5p in sw480 cells. (B) Plate colony formation validated that miR-135a-5p reduced proliferation of sw480 cells; (a) Representative images of plate colony formation; (b) Colony formation rate of sw480 cells. (C) Wound-healing assay showed miR-135a-5p inhibited the migration ability of sw480 cells. (D) Wound healing rate of sw480 cells transfected with miR-135a-5p mimics or NC-mimics. (E) ERp29 was downregulated by miR-135a-5p mimics in sw1116 cells. (F) Plate colony formation validated that miR-135a-5p reduced proliferation of sw1116 cells; (a) Representative images of plate colony formation; (b) Colony formation rate of sw1116 cells. (G) Wound-healing assay showed miR-135a-5p inhibited the migration ability of sw1116 cells. (H) Wound healing rate of sw1116 cells transfected with miR-135a-5p mimics or NC-mimics. (I) Validation that miR-135a-5p inhibited the migration ability of sw1116 cells by transwell chamber migration assay; (a) Representative images of transwell chamber migration assay; (b) Numbers of sw1116^miR-135-5p^ or sw1116^NC-mimics^ cells on the lower surface of the chambers. **P<0.05, **P<0.01, ***P<0.001*.

**Supplementary Figure 2**

**
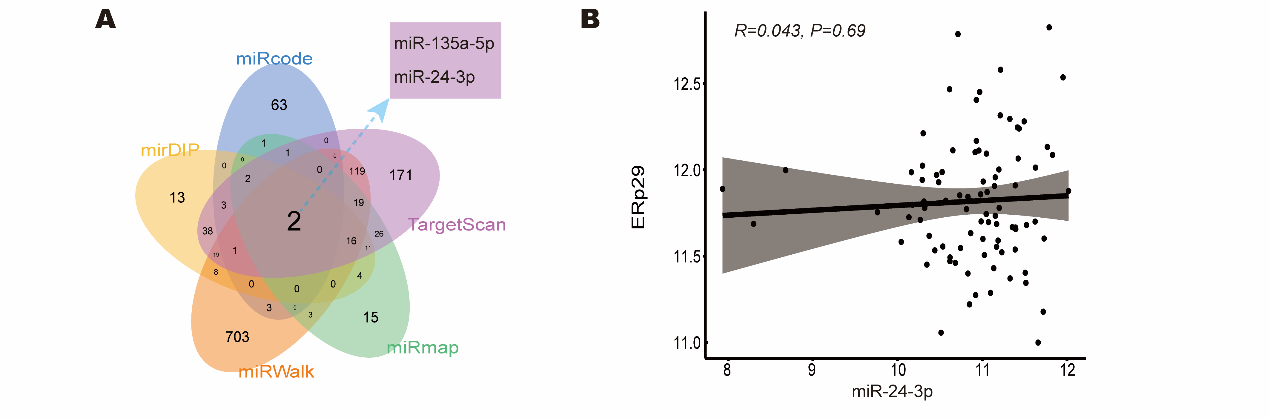
**

**Supplementary Figure 2. Prediction of miRNAs targeting ERp29 using public databases**

(A) Venn diagram analysis of predicted miRNAs targeting ERp29 in five miRNA databases, including miRcode, TargetScan, miRDIP, miRWalk and miRmap, which identified miR-135a-5p and miR-24-3p as candidate miRNAs. (B) Spearman correlation analysis of ERp29 expression and miR-24-3p level in CRC (*P=0.69*).

**Supplementary Figure 3**

**
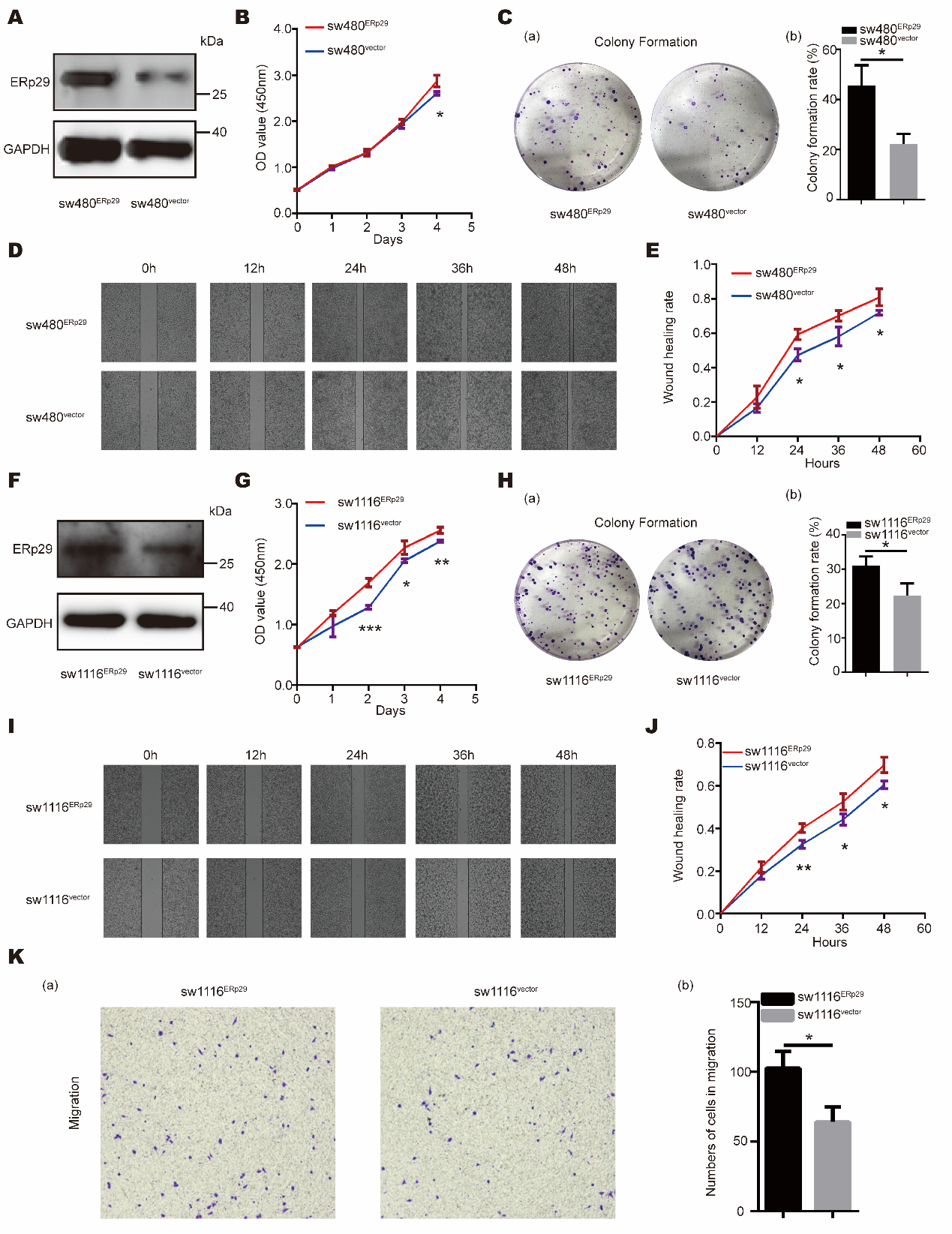
**

**Supplementary Figure 3. ERp29 facilitates proliferation and migration of CRC cells**

(A) Western blot analysis was performed to measure ERp29 expression in sw480 cells transfected with vectors for overexpressing ERp29 or corresponding control vectors. (B) Overexpression of ERp29 promoted cell proliferation as measured by CCK8 assay in sw480 cells. (C) sw480^ERp29^ cell lines formed more cell colonies than control cells; (a) Representative images of plate colony formation; (b) Colony formation rate of sw480 cells. (D) ERp29 enhanced the migration ability of sw480 based on wound-healing assay. (E) Wound healing rate of sw480^ERp29^ and sw480^vector^ cells. (F) Western blot analysis was performed to measure ERp29 expression in sw1116 cells transfected with vectors for overexpressing ERp29 or corresponding control vectors. (G) Overexpression of ERp29 promoted the proliferation ability of sw1116 cells as measured by CCK8 assay. (H) sw1116^ERp29^ cell lines formed more colonies than control cells; (a) Representative images of plate colony formation; (b) Colony formation rate of sw1116 cells. (I) ERp29 enhanced the migration ability of sw1116 cells as determined by wound-healing assay. (J) Wound healing rate of sw1116^ERp29^ and sw1116^vector^ cells. (K) ERp29 promoted the migration ability of sw1116 cells as determined by transwell chamber migration assay; (a) Representative images of transwell chamber migration assay; (b) Number of sw1116^ERp29^ or sw1116^vector^ cells on the lower surface of the chambers. **P<0.05, **P<0.01, ***P<0.001.*

**Supplementary Figure 4**

**
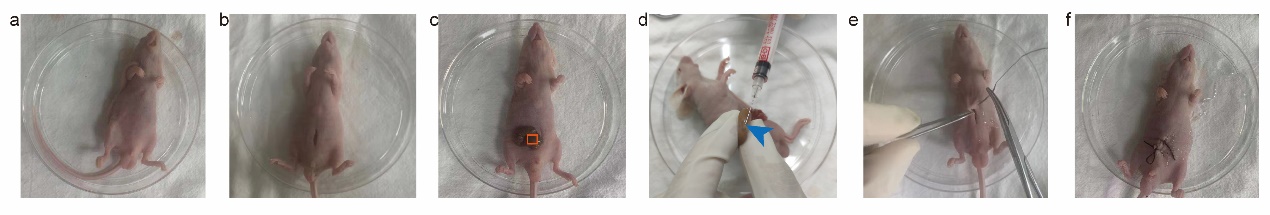
**

**Supplementary Figure 4. Orthotopic xenograft mouse model of CRC**

(a) The mouse was placed in a dish under general anaesthetic. (b) An incision was made through the skin. (c) The cecum was exteriorized, with the orange box indicating the area chosen for transplantation. (d) DLD-1 cells were injected into the cecal walls, with the blue arrow pointing out the location of injection. (e) The opened abdomen was closed by stitching skin and abdominal wall. (f) The suture was ended with the tightened reef knots.
